# Impact of Isonicotinic Acid Blending in Chitosan/Polyvinyl Alcohol on Ripening-Dependent Changes of Green Stage Tomato

**DOI:** 10.3390/polym15040825

**Published:** 2023-02-07

**Authors:** Mohamed A. Taher, Elsherbiny A. Elsherbiny

**Affiliations:** 1 Agricultural Chemistry Department, Faculty of Agriculture, Mansoura University, Mansoura 35516, Egypt; 2 Plant Pathology Department, Faculty of Agriculture, Mansoura University, Mansoura 35516, Egypt

**Keywords:** tomato, shelf-life, chitosan, isonicotinic acid, pigmentation, browning enzymes

## Abstract

The effect of isonicotinic acid (INA) in a chitosan (CS)/polyvinyl alcohol (PVA) blend on ripening-dependent changes of preserved green tomatoes (*Solanum lycopersicum* L.) was examined at room temperature. The results showed that CS/PVA/INA 0.5 mM and CS/PVA/INA 1.0 mM formulations retarded firmness loss and delayed the pigmentation parameters i.e., lycopene (LYP), total carotenes (TCs), and titratable acidity (TA). The CS/PVA/INA 0.5 mM and CS/PVA/INA 1.0 mM formulations were able to delay the increase in malondialdehyde (MDA) and total polyphenol (TP) contents. Furthermore, the peroxidase (POD), polyphenoloxidase (PPO), and phenylalanine ammonia-lyase (PAL) activities of tomatoes coated with CS/PVA/INA 0.5 mM and CS/PVA/INA 1.0 mM formulations were lower than those in other treatments. Meanwhile, the CS/PVA blend had the highest TP content, as well as the highest PPO and PAL activities, at the late stage of maturation. The UV analysis showed that the CS/PVA/INA blend film is a promising UV-protective food packaging material. The pure CS, PVA, and INA formulations, as well as the CS/PVA, CS/PVA/INA 0.5 mM, and CS/PVA/INA 1.0 mM formulations, were characterized by infrared (FTIR). The three polymer formulations showed strong antifungal activity against *Alternaria alternata* and *Botrytis cinerea*.

## 1. Introduction

Tomato is one of the most common vegetable crops of the *Solanaceae* family, and it retains several valuable health constituents including phenolic acids, flavonoids, ascorbic acid, lycopene, and cyclic carotenoids [1]. It is an important fruit cultivated globally all year round for cooking, salad making, and fabricating a wide range of products such as ketchup, juices, and dried powders [1]. However, tomato matures rapidly and decays in the summer season under the impact of high temperatures. Likewise, poor postharvest conditions cause an excessive decline in quality limits such as weight loss and the loss of aromatic constituents [1]. To delay its early deterioration and extend the postharvest period, green-stage tomato fruits are mostly harvested and kept in controlled conditions to decrease postharvest decay [1,2]. Moreover, the susceptibility of tomatoes to fungal pathogens in the postharvest period during the summer season accumulates a huge level of reactive oxygen species (ROS) in their tissues, leading to the oxidation of all types of phenolics, which accelerates their decay [2,3]. The high load of ROS initiates membrane lipid peroxidation (LPOX), which can lead to continuous injury to the cell membrane [4]. Accumulating antioxidants with polyphenolic moieties in plant tissues can resist stress, while their oxidation results in a common browning phenomenon in those tissues [5]. In the last decade, the use of polymer coatings has largely increased to expand the selective permeability of the water/gas interface and, therefore, control the respiratory degree and delay undesirable changes such as aging and anaerobic fermentation, as well as inhibit the growth of microorganisms responsible for fruit spoilage [4,6].

Chitosan (CS) is a natural nontoxic biodegradable polymer with antifungal and antioxidant potential, and it has several applications including in fruit preservation [5,7,8]. Therefore, this biopolymer and its derivatives can be used as natural fungicides. The poor mechanical strength of CS restricts its application [9]. Therefore, blending it with other polymers exhibiting good mechanical strength is an ideal approach to expanding the film properties of CS. In this context, coatings containing CS blended with polyvinyl alcohol (PVA) alone or incorporated with other bioactive ingredients have recently been demonstrated to favorably extend the postharvest period of different fruits [9,10]. Niacin, which is commonly recognized as nicotinic acid, is a water-soluble vitamin that is bio-transformed into nicotinamide adenine dinucleotide (NAD) in the body [11]. NAD is a major coenzyme in redox reactions producing energy. Compounds related to the niacin moiety have many biological effects such as a reduction in serum cholesterol, induction of apoptosis, and antiradical activity [11,12,13]. Isonicotinic acid (INA) or pyridine-4-carboxylic acid is an isomer of niacin. Unlike niacin, it has no ability to support the growth of *Staphylococcus aureus* [14]. The fungistatic properties of pyridine carboxylic acids other than INA have been reported [15]. However, Chakraborty et al. [16] showed that INA at a concentration of 2.5 mM had a strong ability to induce defense enzymes, as well as phenolic constituents, in tomato leaves.

So far, no studies have focused on the effects of the CS/PVA blend on green tomato fruits. Furthermore, the impact of INA on postharvest fruits has not yet been described. Consequently, the current study is aimed at investigating the effect of CS/PVA, CS/PVA/INA 0.5 mM, and CS/PVA/INA 1.0 mM composites on the ripening-dependent changes of green-stage tomatoes, as well as characterizing these biodegradable films, along with an evaluation of their antifungal activity.

## 2. Materials and Methods

### 2.1. Chemicals

The following chemicals were obtained from Sigma-Aldrich (St. Louis, MO, USA): isonicotinic acid (INA), ascorbic acid, polyvinylpolypyrrolidone (PVPP), thiobarbituric acid, Folin–Ciocâlteu reagent, gallic acid, ethylenediaminetetraacetic acid (EDTA), guaiacol, catechol, phenylalanine, and 2,6-dichlorophenol indophenol (DCPIP). Chitosan (CS) (MW: 71.3 kDa, degree of deacetylation: 94%) and polyvinyl alcohol (PVA; degree of polymerization: 1700–1800, 98% hydrolyzed) were obtained from Merck (Darmstadt, Germany). Acetic acid, potassium hydroxide, hydrochloric acid, acetone, petroleum ether (40–60 °C), butylated hydroxytoluene (BHT), ethanol, methanol, hexane, sodium carbonate, sodium bicarbonate, trichloroacetic acid, phosphate salts for buffers, boric acid, and hydrogen peroxide were purchased from the El-Gomhoria Company (Cairo, Egypt) for pharmaceutical chemicals, Cairo, Egypt.

### 2.2. Tomatoes

Agyad 7 genotype tomato fruits were cultivated on a farm near Mansoura, Egypt. Tomato fruits with an index of two colors were harvested at the green phase of maturity, i.e., green fruits with few yellowish spots. Mechanically injured or infected fruits were discarded. Random fruits with uniform shapes and sizes were chosen for the experiment.

### 2.3. Coating Solutions

A 1% solution of PVA was prepared by dissolving 5 g of PVA in 500 mL of distilled water and stirring for 2 h at 60 °C for complete solvation. Then, 5 g of CS was dissolved in 500 mL of 1% (*v*/*v*) acetic acid solution for 6 h under magnetic stirring. Next, the CS/PVA blend (1000 mL) was obtained by mixing the aforementioned solutions for 3 h under magnetic stirring at 70 °C. Isonicotinic acid (INA) was loaded into the CS/PVA blend at different concentrations in 1000 mL of the solution under magnetic stirring for 4 h at 25 °C.

### 2.4. Characterization of Biodegradable Films

#### 2.4.1. Preparation of Samples

First, 5 mL of each film-forming solution was immediately collected in a separate vial and stored at 4 °C until UV/visible analysis. Furthermore, 15 mL of each polymer blend solution was placed in the square section of a Petri dish to a thickness of about 60 µm. Once poured, the blends were left to dry to a constant weight at 45 °C in an incubator. Then, the polymer coatings were peeled from the support and powdered.

#### 2.4.2. UV/Visible Scanning Spectrophotometry

The absorbance of CS/PVA and CS/PVA/INA 1.0 mM solutions was detected at a wavelength of 200–800 nm using a Jenway 7305 UV/visible scanning spectrophotometer (Stone, UK).

#### 2.4.3. Fourier-Transform Infrared Analysis (FT-IR)

For identification of the functional groups and/or potential interactions in pure CS, PVA, and INA, as well as the dry powders of mixed solutions of CS/PVA and CS/PVA/INA 1.0 mM, 1 mg of each sample was mixed with 250 mg of potassium bromide (KBr) powder [4]. The thin pellets were prepared by pressing with a hydraulic pellet press and then subjected to Fourier-transform infrared spectrophotometry (FTIR, Thermo Scientific Nicolet Is20 FTIR spectrometer, Waltham, MA, USA) in the wavenumber range of 4000–500 cm^−1^ at a resolution of 4 cm^−1^.

### 2.5. Antifungal Activity

The antifungal potential of CS/PVA, CS/PVA/INA 0.5 mM, and CS/PVA/INA 1.0 mM composites against *Alternaria alternata* AUMC 10301 and *Botrytis cinerea* AUMC 6095 (AUMC, Assiut University Mycological Centre, Asyut, Egypt) was assessed in vitro according to the method of Taher et al. [17]. The tested concentration of CS or PVA in the medium was 2 g/L.

### 2.6. Application of Biodegradable Coating Treatments

Selected fruits were quickly washed with sodium hypochlorite (0.05% *w*/*v*) for 3 min, rinsed with sterile water, and then air-dried in a laminar flow cabinet at room temperature [1]. Then, fruits were divided into four groups (40 fruits each); the first group was used as a control, whereby fruits were immersed in distilled water for 5 min. The second group was dipped in a polymer solution of CS/PVA for the same period. The third and fourth groups were separately put into solutions of CS/PVA/INA 0.5 mM and CS/PVA/INA 1.0 mM, respectively, for the same duration. After the dipping process, fruits were dried at room temperature with the aid of continuous airflow. Fruits were then placed in transparent folding PET boxes with 4–5 holes (7–8 mm) to maintain the composition of air within the container (90% relative humidity) for 20 days at 25 ± 3 °C. All treatments in the trial were performed in triplicate. The samples needed for the subsequent analyses were collected at time zero and after 5, 10, 15, and 20 days.

### 2.7. Physical and Chemical Properties

#### 2.7.1. Fruit Firmness

Fruit firmness was recorded on tomato fruits using an Effegi penetrometer supplemented with an 8 mm diameter plunger penetrator, and the results are presented in newtons [18].

#### 2.7.2. Titratable Acidity

TA was estimated using the back-titration protocol written by Taher et al. [1]. The results are presented in mg citric acid·g^−1^ of tissue homogenate. Then, 4 g of tissue was homogenized with 10 mL of potassium hydroxide (0.08 N), and excess KOH was neutralized by hydrogen chloride (0.08 N) to the endpoint of phenol–phthalein (pH 8.2). Titratable acidity was then calculated.

#### 2.7.3. Total Carotenoid Content (TC)

TC content was estimated according to the method of Carvalho et al. [19]. Two grams of tomato was homogenized with acetone (10 mL), and the solid phase was re-extracted with the same volume of acetone until the tissue was colorless. The combined acetone was shifted in a separatory funnel containing 20 mL of petroleum ether (40–60 °C). Distilled water was added slowly to the mixture with shaking. The first two steps were repeated twice with the acetone layer. Carotenoids in petroleum ether were moved over sodium sulfate (anhydrous) to eliminate traces of water. The absorbance of the petroleum ether layer was recorded at 450 nm [1]. The concentration of TCs in each sample was calculated, and the results are expressed in µg·g^−1^.

#### 2.7.4. Extraction and Determination of Lycopene

Extraction and spectrophotometer estimation of lycopene was conducted in triplicate according to the method of Fish et al. [20]. Briefly, 0.4 g of each stirred puree tomato sample was added separately to an amber-colored vial containing 3 mL of 0.05% (*w*/*v*) BHT/acetone, 5 mL of 95% ethanol, and 10.0 mL of hexane. Vial mixtures were then relocated into an orbital shaker to mix at 180 rpm for 15 min. After shaking and residue precipitation, 3 mL of deionized water was added to each vial, and the samples were shaken for another 5 min. Shaken vials were left at room temperature for 5 min to allow for phase separation. Then, the upper layer was collected and treated for dilution with hexane (1:10, *v*/*v*). The lycopene absorbance was recorded at 503 nm. The amount of LYP was calculated using the following formula:

Lycopene content (µg/g) = (A × 31.2 × dilution)/W,

where A is the absorbance of the tested sample at 503 nm, W is the weight of the sample, and 31.2 is a constant derived from the molar extinction coefficient of lycopene in hexane (17.2 × 10^4^ M^−1^·cm^−1^).

#### 2.7.5. Determination of Ascorbic Acid

Ascorbic acid was extracted from tissue homogenates using an oxalic acid solution, and the resultant filtrate was titrated against DCPIP dye solution according to Taher et al. [1]. A solution of ascorbic acid with known concentration was used as the standard. The concentration of ascorbic acid in each sample was calculated, and the results are expressed in mg·g^−1^.

#### 2.7.6. Extraction and Determination of Total Phenolic Content

The extraction and determination of TP content in tomato fruits were conducted using a previously described method [1]. In detail, 5 g of fruit tissue for each treatment was soaked in 25 mL of 80% methanol for 16 h, followed by centrifugation at 5000× *g* for 20 min at room temperature. Then, 0.5 mL of supernatant was well mixed with 0.5 mL of Folin–Ciocâlteu reagent, 2.5 mL of sodium carbonate (2 N), and 1.5 mL of distilled water and then kept at 25 °C for 90 min. The absorbance of each sample mixture was measured at 760 nm. Gallic acid was used to plot the standard curve of total polyphenols in a range of 25–150 µg/mL. The TP contents of tomato are expressed in µg gallic acid equivalent per g fresh weight.

#### 2.7.7. Malondialdehyde (MDA) Content

MDA content was extracted from tomato homogenate using a trichloroacetic acid solution. The mixture was centrifuged, and a known volume of the supernatant was treated with a thiobarbituric acid solution, followed by boiling and centrifugation. The resultant supernatant was measured at 532 nm. The results are defined in µmol of MDA per g fresh weight [21].

### 2.8. Activity of Antioxidant and Browning Enzymes

For polyphenoloxidase (PPO) and peroxidase (POD) assays, 2 g of fruit tissue was homogenized in 10 mL of 100 mM phosphate buffer (pH 6.4) containing 0.2 g of PVPP. The mixture was centrifuged at 10,000× *g* for 40 min at 4 °C, and the supernatants were used as PPO and POD enzyme extracts. The activity of PPO was assessed according to the method of Elsherbiny and Taher [22] by evaluating the enzymatic oxidation of 0.5 mL of 5% (*w*/*v*) catechol using 0.2 mL of enzyme extract after an incubation period of 2 min with 0.05 M phosphate buffer (pH 7.0). The increase in absorbance at 398 nm was automatically documented for 3 min, using a spectrophotometer (UV–1750, Shimadzu, Kyoto, Japan). One unit of PPO activity is defined as a change in OD at 398 nm·min^−1^·mg^−1^ protein. For POD activity, the reaction mixture contained 2 mL of 100 mM phosphate buffer (pH 6.4), 2 mL of 8 mM guaiacol, and 0.1 mL of enzyme extract, and the reaction was started by adding 1 mL of 24 mM H_2_O_2_. The increase in absorbance at 460 nm was recorded [22]. One unit of POD activity is defined as a change in OD at 460 nm·min^−1^·mg^−1^ protein.

Catalase (CAT) activity was estimated according to the method of Luo et al. [23]. Briefly, frozen tomato tissue (2.0 g) was homogenized in 10 mL of 50 mM sodium phosphate buffer (pH 7.8) at 4 °C. Then, centrifugation was performed at 10,000× *g* at 4 °C for 20 min, and the supernatant was considered as the crude enzyme extract for the CAT assay. The reaction mixture (3 mL) contained 400 μL of enzyme extract, 600 μL of H_2_O_2_ (10 mM), and 2 mL of 50 mM phosphate buffer. One unit of CAT activity is defined as a change in OD at 240 nm·in^−1^·mg^−1^ protein.

For phenylalanine ammonia-lyase (PAL) assay, 1 g of tomato tissue was extracted in 5 mL of 200 mM boric acid buffer (pH 8.8) containing 10% (*w*/*v*) polyvinylpyrrolidone, 50 mM β-mercaptoethanol, and 1 mM EDTA according to the method of Assis et al. [24]. The homogenate was centrifuged for 30 min at 12,000× *g*, and the supernatant was used for the estimation of enzyme activity. A volume of 200 μL of the extract was incubated with 1 mL of 20 mM phenylalanine and 2 mL of extracting buffer at 24 °C for 2 min; then, 0.1 mL of 6 M hydrochloric acid was used to stop the reaction, after which the absorbance at 290 nm was recorded. One unit of PAL activity is expressed as a change in OD at 290 nm·min^−1^·mg^−1^ protein. The Lowry assay, with bovine serum albumin (BSA) as the standard, was applied to determine the protein concentration [25].

### 2.9. Statistical Analysis

All data are listed as the mean ± standard error (SE). Turkey’s HSD test was performed using a one-way analysis of variance (ANOVA) in SAS (version 9.1, SAS Institute, Cary, NC, USA) with a *p*-value < 0.05 considered significant [26].

## 3. Results and Discussion

### 3.1. Optical Properties

**Figure 1:**
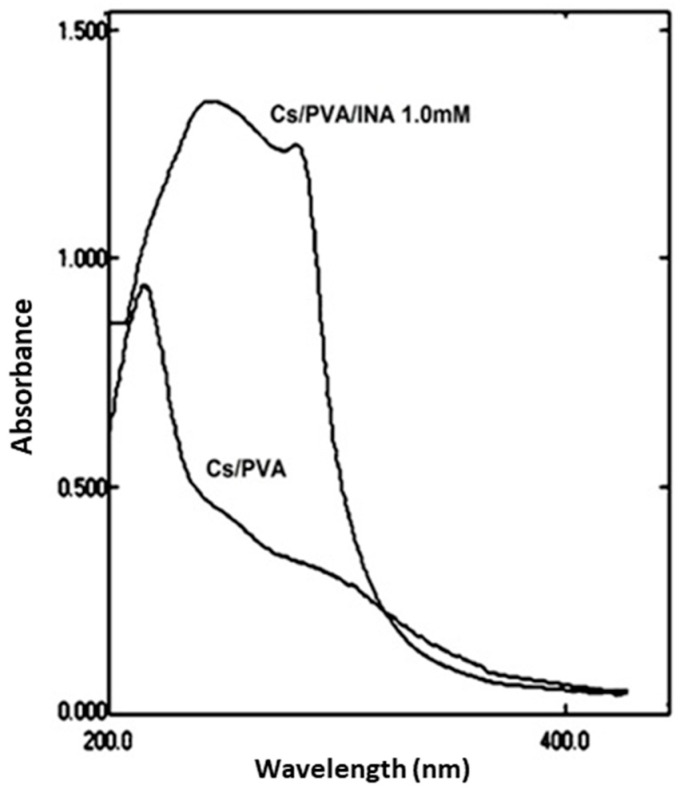
UV spectra of CS/PVA and CS/PVA/INA 1.0 mM composites.

The absorption spectrum of the CS/PVA formulation showed a characteristic peak with a maximum wavelength of 217 nm (Figure 1), revealing the presence of the carbonyl group and the unsaturation of the ethylene group in the unpolymerized vinyl acetate units of PVA; similar results were confirmed by Abdolrahimi et al. [27]. It is well known that pure INA has a characteristic strong absorption peak at 263 nm [28]. The absorbance of INA complexes at higher wavelengths has also been described [28]. In this study, the CS/PVA/INA formulation showed two absorption peaks with maximum values at 242 and 282 nm, which could represent the shifted values of PVA and INA, respectively.

The coordination interaction between CS/PVA and INA yielded a more extensive π → π* conjugated system than the free INA ligand. The absorption intensity of CS/PVA/INA was far stronger than that of CS/PVA, clearly indicating that the coordination to INA increased the absorption intensity of the polymer blend; therefore, the light transmittance was notably reduced in the UV region. In short, CS/PVA/INA composites are promising UV-protective food packaging elements. Nguyen et al. [29] revealed that the incorporation of plant crude extracts in CS films enhanced their UV-blocking ability compared to CS only. Likewise, the semiconductor nanoparticles were effectively used to enhance PVA’s properties in UV light [30].

### 3.2. Fourier-Transform Infrared Analysis (FTIR)

**Figure 2:**
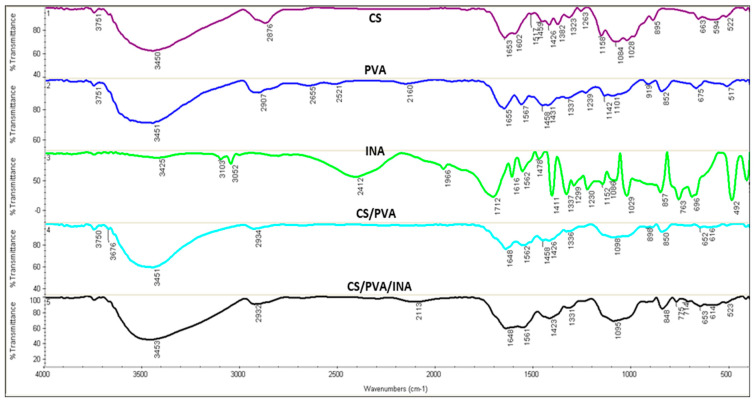
FTIR spectra of chitosan (CS), polyvinyl alcohol (PVA), isonicotinic acid (INA), and CS/PVA, and CS/PVA/INA 1.0 mM blends.

Figure 2 displays the FTIR spectra of pure CS, PVA, and INA, as well as the blends of CS/PVA and CS/PVA/INA 2 mM. The resultant spectra display characteristic bands of vibrations related to the functional groups of the pure compounds or those formed in the prepared blends. The typical peaks of CS were at 3450 cm^−1^ (O–H stretching), 2876 cm^−1^ (C–H stretching) [31], 1652 cm^−1^ (N–H bending), 1324 cm^−1^ (C–N stretching or amide II) [32], and 1085 cm^−1^ (C–O stretching) [33]. The IR spectrum of CS in the present study agrees with those previously noted [34,35]. The PVA exhibited chief absorption peaks at 3452 cm^−1^ (OH stretching), 2907 cm^−1^ (CH stretching), 1431 cm^−1^ (CH bending), and 1101 cm^−1^ (C–O stretching). The peak at 1655 cm^−1^ was assigned to stretching vibrations of C=O groups of the residual vinyl acetate repeat units in the PVA, as 2% of these groups remained unhydrolyzed in the pure material (PVA, 98% hydrolyzed). The IR spectrum of PVA in this study agrees with those previously recorded [36].

The absorption peaks at 763 cm^−1^ and 696 cm^−1^ were related to the deformation vibration of the aromatic ring in INA [37]. The peaks of INA detected at 1616, 1562, 1478, 1411, and 1337 cm^−1^ (Figure 2) are closely parallel to those of picolinic acid observed by Świderski et al. [38]. The previously noted peaks were associated with C=C and C=N stretching from the pyridine ring. The very intense band at 1712 cm^−1^ was due to the presence of stretching vibrations ν (C=O) from the carboxyl group of INA. The band at 3052 cm^−1^ was related to νs (CH) vibration of ring protons in the molecule. It is well known that broad absorption bands between 2607 and 2152 cm^−1^ indicate the existence of an O–H⋅⋅⋅N type of intermolecular hydrogen bonding [37,39]. Hence, the peak found at 2412 cm^−1^ could be related to the presence of this type of intermolecular hydrogen bonding between INA molecules.

**Figure 3:**
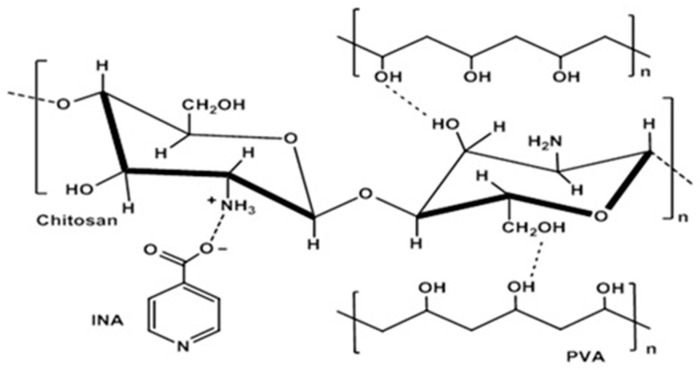
Scheme of the possible interactions linking chitosan (CS), polyvinyl alcohol (PVA), and isonicotinic acid (INA).

The FTIR spectrum of the CS/PVA blend displayed the chemical interactions between both components. The band near 3452 cm^−1^ related to OH stretching of PVA was broadened and shifted toward a lower frequency. The band at 1652 cm^−1^ of N–H bending was shifted to a slightly lower wavenumber (4 cm^−1^ lower than the CS) in the CS/PVA composite. The FTIR spectrum of the CS/PVA/INA blend was largely identical to that of the CS/PVA blend with some differences. For example, the appearance of the absorption peaks related to the deformation vibration of the pyridine ring of INA at slightly higher wavelengths of 775 cm^−1^ and 713 cm^−1^ could confirm that the pyridyl N atom was not coordinated. The nonappearance of absorption bands around 1700 cm^−1^ in the CS/PVA/INA formulation revealed that the COOH groups of the ligand of INA were deprotonated; a similar observation was reported [40,41]. The previous observations support the role of INA carboxylate in the formation of ionic crosslinking bonds with amino groups of CS. Likewise, the absence of the broad absorption band at 2412 cm^−1^ related to the O–H⋅⋅⋅N type of intermolecular hydrogen bonding of INA in the CS/PVA/INA formulation supported the potential role of INA carboxylate in the formation of new linkages. Overall, the formation of CS/carboxylic acid complexes via moderate ion crosslinks and hydrogen bonding was determined [42]. Figure 3 shows the proposed structure of CS/PVA/INA composites.

### 3.3. Antifungal Activity

**Figure 4:**
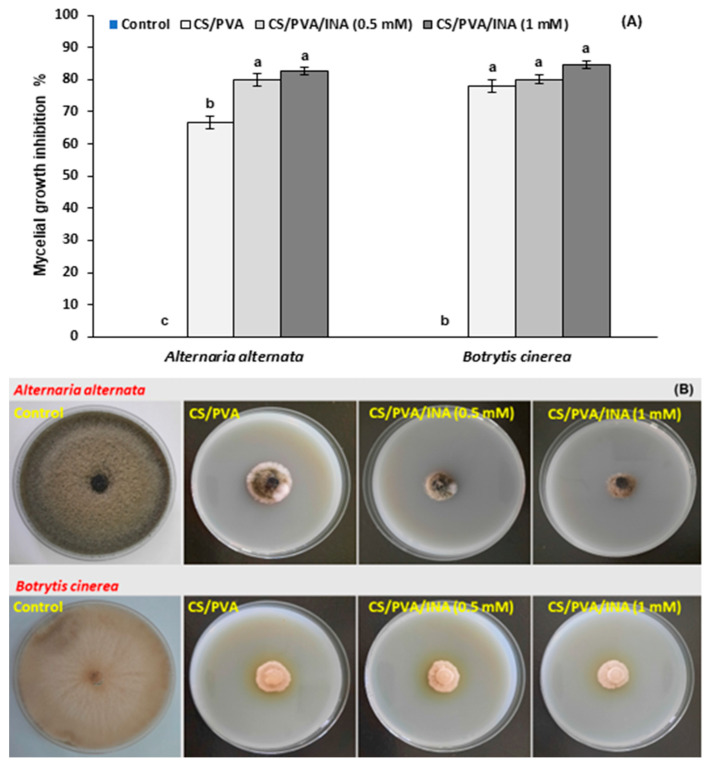
Effect of CS/PVA, CS/PVA/INA 0.5 mM, and CS/PVA/INA 1.0 mM composites on (**A**) mycelial growth inhibition (%) and (**B**) radial growth on PDA plates of *Alternaria alternata* and *Botrytis cinerea*. Results represent the mean ± standard error (SE). Bars with different letters are significantly different according to Tukey’s HSD test at *p* < 0.05.

The antifungal activity of CS/PVA, CS/PVA/INA 0.5 mM, and CS/PVA/INA 1.0 mM composites was evaluated using the radial growth method against *A. alternata* and *B. cinerea*. A significant reduction in the mycelial growth of both pathogens was detected in the CS/PVA, CS/PVA/INA 0.5 mM, and CS/PVA/INA 1.0 mM composites on the fifth day of incubation compared with the control group (Figure 4A,B). In the case of *A. alternata*, CS/PVA, CS/PVA/INA 0.5 mM, and CS/PVA/INA 1.0 mM composites recorded inhibition zones of 66.7%, 79.9%, and 82.6%, respectively. The same composites caused a voluminous inhibition in the mycelial growth of *B. cinerea* by 77.8%, 80.1%, and 84.5%, respectively. Noteworthily, INA in the polymer blend significantly increased the inhibition percentages against *A. alternata* in comparison with the CS/PVA blend and the control.

The mode of action depends on the electrostatic interactions between CS and the negatively charged moieties of phospholipids found in the cell membrane of fungi [43,44]. When the cell membrane is disrupted, CS can penetrate the cell and suppress the synthesis of DNA, RNA, and related proteins. Several reports have evaluated the antifungal and antimicrobial activity of INA synthetic derivatives [43,45], as well as the fungistatic properties of pyridine carboxylic acids other than INA [15]. As far as we know, this is the first study to establish the antifungal activity of a CS/PVA composite alone or incorporated with INA against the major postharvest pathogens of tomato fruit.

### 3.4. Physical and Chemical Properties of Tomatoes

#### 3.4.1. Fruit Firmness

**Figure 5:**
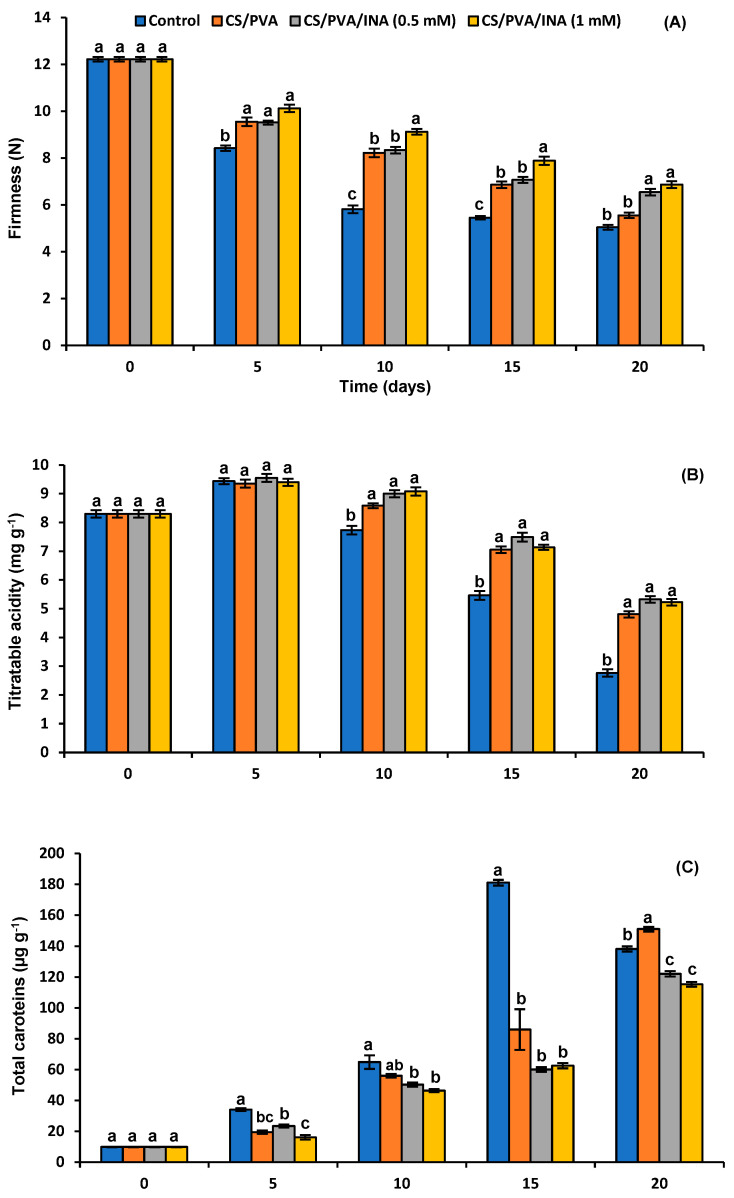

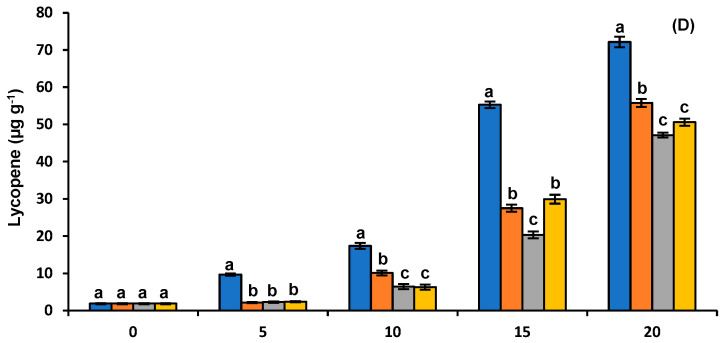
Effect of coating treatments on (**A**) firmness, (**B**) titratable acidity, (**C**) total carotenes, and (**D**) lycopene in tomato fruit during the shelf-life period. Results represent the mean ± standard error (SE). Bars with different letters are significantly different according to Tukey’s HSD test at *p* < 0.05 for the same time point.

The firmness of the fruits under study significantly decreased over the storage period for both the control and the coated samples (Figure 5A). There was a sharp decrease in firmness in the control group, which had the significantly lowest values until the 15th day of storage. Correspondingly, it also had the lowest value of firmness by 5.04 at the end of storage. CS/PVA/INA 1.0 mM recorded the highest values in firmness throughout the experiment, reaching 6.86 N at the end of the experiment (Figure 5A). Tomato is a climacteric fruit whose firmness is reduced with the progress of ripening, which is associated with its short postharvest period [46]. This fruit softening occurs as a result of the enzymatic degradation of cell wall components [4]. As the softening progresses, depolymerization of pectin substances and hemicelluloses happens simultaneously with the promotion of the activities of related enzymes such as pectinesterase, resulting in cell wall disruption and disintegration [46]. A loss of firmness was rapidly presented by the uncoated fruits, which might be due to the degradation of the cell wall and the appearance of related changes.

Edible biodegradable films preserve firmness by diminishing the respiration process, delaying ripening, decelerating senescence, and reducing cell wall degradation. The presence of the INA elicitor in the CS/PVA mixture sustained a higher level of fruit firmness in contrast to the untreated samples and slowly decreased their firmness until the end of storage. In this respect, increasing the efficiency of polymer blends to retain more firmness in fruits via their combination with salicylic acid was reported [4].

#### 3.4.2. Titratable Acidity

The TA of treated and uncoated tomatoes was reduced with shelf-life. Principally, our results displayed that control fruits had a significantly lower TA content than those immersed in CS/PVA formulations from the 10th day until the end of storage (Figure 5B), indicating that CS/PVA-based blends retarded maturation by providing a bioactive transparent biofilm around the surface of tomato fruit. The TA contents of the control, CS/PVA, CS/PVA/INA 0.5 mM, and CS/PVA/INA 1.0 mM treatments were 2.76%, 4.80%, 5.32%, and 5.22% on the last day of the experiment period, respectively (Figure 5B).

The increase in TA after the fifth day of storage for all groups was mostly ascribed to the accumulation of organic acids (OAs) and the subsequent reduction to different degrees can be attributed to ripening rates of higher and respiration, where OAs could be expended as a substrate consumed in respiration reactions or in their transformation to sugars [47]. In our study, the CS/PVA film enriched with INA was the best treatment in preventing respiration during the storage period, thus decreasing the use of OAs and, consequently, increasing the content of TA. Shehata et al. [48] also reported a similar result of TA alteration, establishing that the acidity depletion was lower in breaker-stage fruits treated with the CS 0.5% (*w*/*v*) film compared to the control group. Moreover, the delaying effect of nano-silicon oxide in chitosan film on tomato fruit respiration was described [5] explaining the delayed depletion of OAs, leading to elevated TA values. Furthermore, the decreased loss of TA of orange-stage tomatoes using a Cu/chitosan nano-net was reported [49]. On the contrary, our results differ from those obtained by Chrysargyris et al. [50], who concluded that *Aloe vera* gel had no significant impact on the TA of tomatoes after 7 and 14 days of storage. Predominantly, this study showed that the CS/PVA/INA 0.5 mM and CS/PVA/INA 1.0 mM formulations had the highest values of TA throughout the experimental period, indicating that INA plays a potential role in delaying the ripening of tomato fruits. In this respect, the inhibition of ethylene production by its isomers picolinic acid and pyrazinamide was reported in [51]. Moreover, CS was able to suppress the respiratory rate and the degradation of chlorophyll [52].

#### 3.4.3. Total Carotenoids

Figure 5C shows the alternations in the TC content of tomatoes covered with different CS/PVA formulations throughout the trial period at room temperature. The extent of TCs gradually increased with significance and reached a maximum (181.03 µg/g FW) in uncoated fruits after 15 days of storage, before intensely declining on the 20th day to 138.23 µg/g FW. Tomato fruits treated with CS/PVA/INA 0.5 mM and CS/PVA/INA 1.0 mM formulations exhibited a slight but contentious increase in TCs until the 15th day, followed by a notable increase at the end of the trial period with values of 122.13 and 115.35 µg/g, respectively. The polymer blends containing INA had significantly lower TC values than the control throughout the experiment period. Moreover, a reasonable rate of increase in TC content was noticed in CS/PVA throughout the storage period.

During fruit maturity, the cyclization enzymes associated with the biogenesis of cyclic carotenoids are strongly induced. The maximum velocity of these enzymes was reported to be in green-stage tomato fruit [53]. On the other hand, the highest TC content, mostly consisting of β-carotene and lycopene, was found to exist in ripe tomatoes [54]. In the present work, the gradual elevation of TCs until the 15th day, followed by the huge reduction in uncoated fruits, proved their further maturity compared to those immersed in different CS/PVA formulations; a similar observation was reported [1,50]. For instance, untreated tomatoes reached the significantly greatest value of TCs after 15 days when compared with those coated with different formulations based on gum Arabic (GA) [1]. The significant reduction in TCs in the CS/PVA treatment when compared to control uncoated fruits on the 15th day indicated the effectiveness of the polymer blend in retarding maturation to some extent. This outcome is generally in accordance with Ali et al. [55], who found that GA reduced the progress of maturation by diminishing the respiration degree and, thus, preserving high content of TCs for a longer time compared to uncoated fruits. Similarly, Taher et al. [1] found that the TC content of GA/PVP-covered tomato fruits was significantly lower than that of uncoated fruits until the 15th day of storage. The small continuous increase in TC content of CS/PVA/INA 0.5 mM and CS/PVA/INA 1.0 mM until the end of the trial in this study reflects the probable role of INA in delaying the progression of ripening. To our knowledge, no previous studies focused on the impact of INA on the pattern of pigmentation of tomato fruit.

#### 3.4.4. Lycopene

Lycopene (LYP) content increased throughout postharvest storage in all tested samples (Figure 5D). Lycopene levels elevated slowly in all tested groups until the 10th day. followed by a huge increase, particularly in uncoated fruits, which remained significant until the end of the storage period. They reached the maximum value of 72.16 µg/g for uncoated samples on the last day of the trial period. Inversely, the CS/PVA/INA 0.5 mM-treated fruits had the significantly lowest values of LYP after the 15th and 20th days of the storage period at 20.32 and 47.13 µg/g FW, respectively. Moreover, a reasonable increase in LYP content was detected in the CS/PVA and CS/PVA/INA 1.0 mM-treated fruits with values of 55.78 and 50.62 µg/g FW, respectively, at the end of the storage period.

The appearance of the distinctive red color of the fruit is principally associated with the advanced biosynthesis of LYP through ripening [56]. An increased LYP content in tomatoes occurs upon inducing the activities of the enzymes catalyzing its biosynthesis and reducing the levels of the cyclase enzymes responsible for further biosynthetic metabolism of LYP. The evidence of rapid ripening of control fruits, including the continuous increase in LYP concurrently with the sharp decrease in TCs at the end of the postharvest period, could be mostly explained by the decreased activation rate of cyclase enzymes. On the other hand, the continuous increase in LYP and TC contents in tomatoes coated by CS/PVA formulations reflected a slow rate of ripening. Overall, the genes encoding lycopene biosynthesis enzymes were upregulated, while cyclase genes related to the biogenesis of cyclic carotenes from the LYP precursor were downregulated in tomatoes at the breaker stage [53].

INA and picolinic acid are structural isomers of nicotinic acid. In our study, the slow degree of pigmentation in tomatoes treated with the polymer blend enriched with INA reflected the retardation of ripening processes, possibly due to the inhibition of ethylene production. To the best of our knowledge, no previous reports evaluated the impact of INA on the postharvest characteristics of fruits. However, the inhibition of ethylene production by its isomer picolinic acid and pyrazinamide was reported [51]. Picolinic acid can bind at the active site of 1-aminocyclopropane-1-carboxylicacidoxidase, preventing the enzyme from interacting with its biological substrates and, therefore, blocking ethylene biosynthesis.

#### 3.4.5. Ascorbic Acid Content

**Figure 6:**
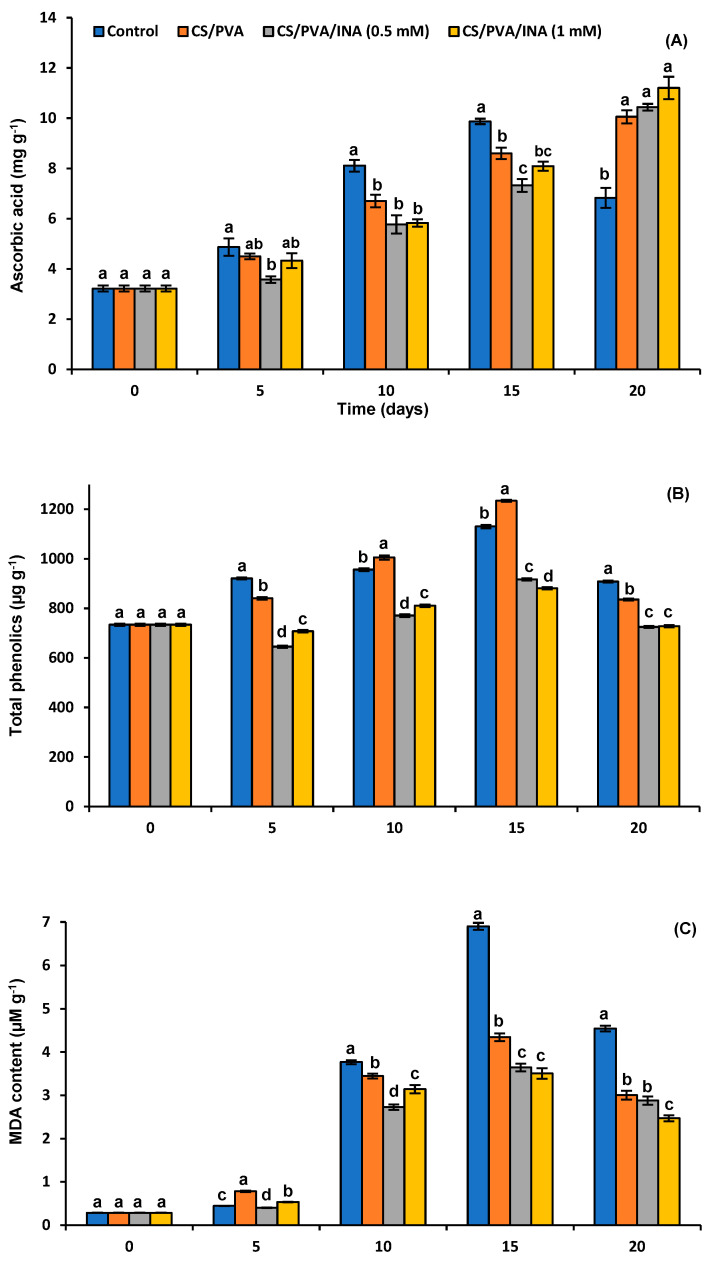
Impact of polymer coating treatments on (**A**) ascorbic acid, (**B**) total phenolic, and (**C**) malondialdehyde (MDA) levels in tomato fruit during the shelf-life period. Results represent the mean ± standard error (SE). Bars with different letters are significantly different according to Tukey’s HSD test at *p* < 0.05 for the same time point.

The ascorbic acid (AA) content of tomato fruit gradually increases until the fruit reaches full maturity, followed by a notable decrease [44]. Figure 6A shows that the AA content progressively increased in uncoated tomato fruits and attained the maximum peak (9.87 mg/g) on the 15th day, before declining to 6.82 mg/g. Hence, the significant decrease in AA content at the end of the storage period reflected the over-maturity of uncoated fruits. Application of CS/PVA, CS/PVA/INA 0.5 mM, and CS/PVA/INA 1.0 mM treatments resulted in significantly lower values when compared with the control after 10 and 15 days of storage, followed by significantly higher values at the end of the storage period of 10.05, 10.43, and 11.20 mg/g FW, respectively. Our results show that all coating treatments prevented the deterioration of AA content related to full-stage maturity. A similar delayed decrease in the AA content of coated tomatoes was indicated in [1,50,55].

It is well known that phenolic antioxidants have a protective effect on AA [57]. In this respect, the authors of [1,58,59] established that edible coatings supplemented with plant extracts rich in phenolics and other antioxidant ingredients were able to preserve higher AA content in tomato fruits. However, it seems that no readily available information exists about the impact of INA on AA. Interestingly, the radical-scavenging activities (RSA) of pyridine carboxylic acids and their hydrazides have been reported [12]. The significantly higher values of AA at the end of the storage period in CS/PVA/INA 0.5 mM and CS/PVA/INA 1.0 mM treatments (Figure 6A) could be ascribed to the protective role of INA on AA content, concurrently with maturity retardation.

#### 3.4.6. Total Polyphenols

In the control group, the TP content in tomato fruits during storage increased intensely in the first 5 days, followed by a minor increase in the next 5 days, a huge increase on the 15th day, and a drop on the 20th day of the trial (Figure 6B). In CS/PVA-coated fruits, the TP content showed the significantly highest value at 1234.32 µg GAE /g on the 15th day of storage in comparison to the other treatments. On the other hand, the TP level in CS/PVA/INA 0.5 mM- and CS/PVA/INA 1.0 mM-treated fruits displayed significantly (*p* < 0.05) lower values in comparison with the CS/PVA-coated and uncoated tomato fruits throughout the experiment.

Edible polymer films increase the quality attributes of tomatoes by decreasing the moisture loss and gas exchange while avoiding pathogenic fungal attacks and related deterioration [60]. The accumulation of plant TPs increases during different types of stress conditions, such as injury and pathogenic attack [18]. The quantity of TPs can be used as an additional indicator of enzymatic antioxidants as a defensive response in different plants against environmental stress. The CS/PVA blend resulted in clear increases in TP content, particularly in the late stage of storage; a similar observation was reported in [1]. They found that the formulations based on GA and PVP showed significantly higher TP content than that of untreated tomato fruits at the end of the trial. The previous result mostly disagrees with the findings of Dávila-Aviña et al. [61], who found that Carnauba and mineral oil coatings significantly reduced the TP, flavonoid, and LYP contents than control fruits when applied on fresh tomatoes at the breaker and pink maturity stages.

INA in the CS/PVA blend (CS/PVA/INA 0.5 mM and CS/PVA/INA 1.0 mM formulations) resulted in different responses, with significant diminutions in TP content throughout the storage period. Similarly, the accumulation of TPs in green tomatoes coated with a nano-SiOx chitosan film was lower compared to uncoated fruits, as described by Zhu et al. [5]. However, the application of INA on tomato leaves increased the accumulation of phenolics after 24 h of treatment [16]. This discrepancy may be elucidated by the differences in the physiology of the treated part of the plant, the concentration of INA used, and the presence or absence of the coating film.

#### 3.4.7. Malondialdehyde (MDA)

MDA is the final product of LPOX induced by ROS, and its huge increase is an indicator of oxidative stress due to the degradation of cell membranes. The present study presents the degree of LPOX assessed as µmol of MDA per g tissue (Figure 6C). The MDA level reached the significantly highest value of 6.9 µmol of MDA per g FW for uncoated samples on the 15th day of storage. In contrast, its level was 4.34, 3.64, and 3.50 µmol of MDA per g FW for the CS/PVA, CS/PVA/INA 0.5 mM, and CS/PVA/INA 1.0 mM treatments in the same period, respectively. Overall, all coating treatments led to significantly (*p* < 0.05) lower values of MDA than the control uncoated fruits, beginning from the 10th day until the end of the storage period. Our findings concerning lipid peroxidation showed that CS/PVA/INA is a powerful tool for retarding postharvest oxidative damage. Coating postharvest fruits with biodegradable polymer alone or enriched with bioactive additives significantly decreases the level of MDA, thus preserving the functions of cellular membranes and diminishing the cell permeability levels [62].

Overall, the formulations based on CS/PVA could protect tomato fruit from environmental stress. This could be attributed to the capacity of the coating film to generate a barrier to the O_2_ needed for LPOX, thereby maintaining membrane integrity [62]. The powerful role of different bioactive ingredients incorporated in polymer coatings such as nano-SiOx, as well as ascorbic and salicylic acids, in reducing LPOX in different postharvest fruits was reported in [5,9]. To our knowledge, no documents have studied the effect of INA on the production of ROS and LPOX in postharvest fruits. However, its application on tomato leaves at a concentration of 2.5 mM significantly reduced ROS production and LPOX [16]. However, higher concentrations of INA led to stress conditions in the plant, with an extremely high accumulation of MDA. Interestingly, the antiradical potential of niacin-related compounds including INA was reported [12]. Moreover, exogenous niacin treatment increased stress tolerance in kiwifruit via various mechanisms including the activation of NADPH oxidases [63]. It was also reported that PiA spray stimulated antioxidative enzymes and reduced MDA production [64].

### 3.5. Antioxidant and Browning Enzymes

**Figure 7:**
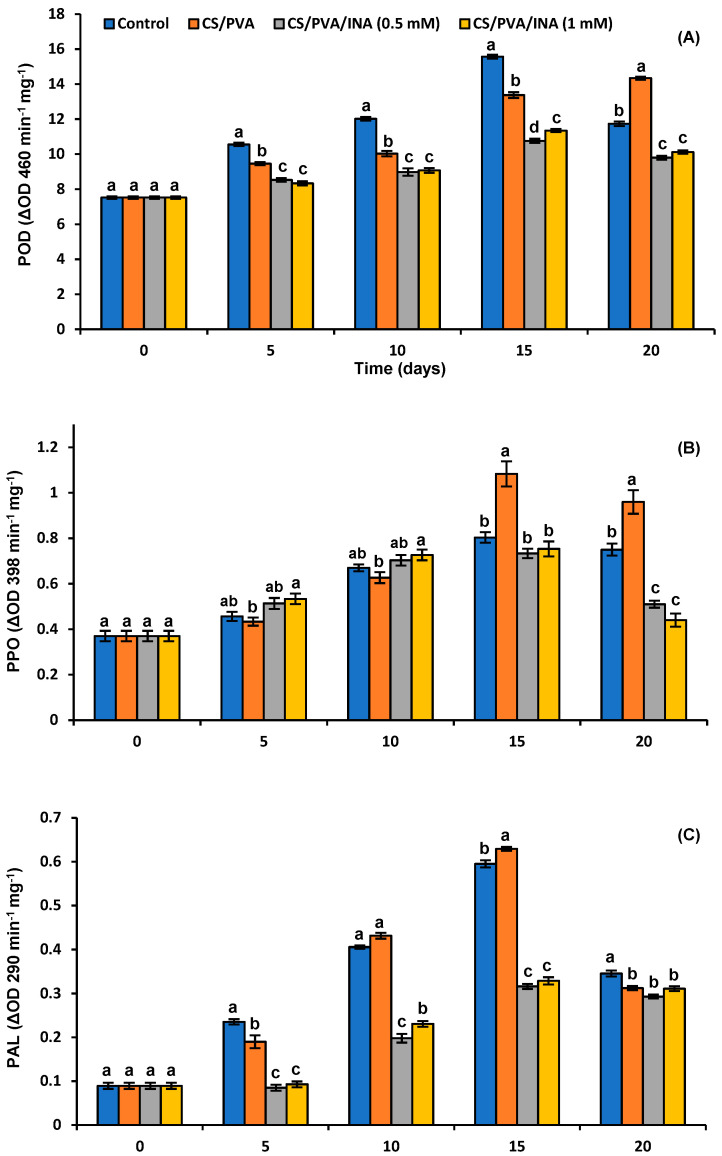

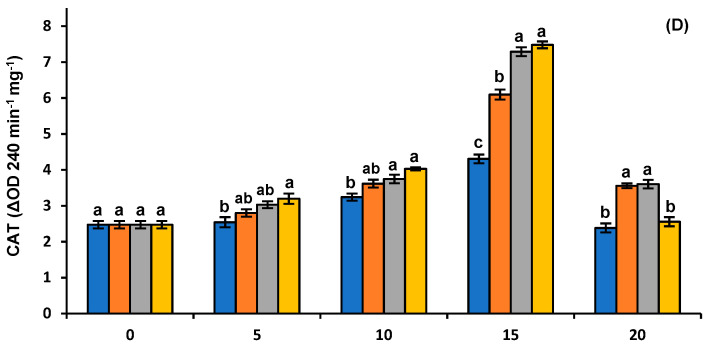
Impact of polymer coating treatments on enzymes activity of (**A**) peroxidase (POD), (**B**) polyphenoloxidase (PPO), (**C**) phenylalanine ammonia-lyase (PAL), and (**D**) catalase (CAT) in tomato fruit during the shelf-life period. Results represent the mean ± standard error (SE). Bars with different letters are significantly different according to Tukey’s HSD test at *p* < 0.05 for the same time point.

The peroxidase (POD) activity reached the peak on the 15th day in the control group, with the significantly highest value of 15.57 U, and then decreased at the end of storage (Figure 7A). Similarly, the CS/PVA/INA 0.5 mM and CS/PVA/INA 1.0 mM formulations reached their peaks on the same day with significantly lower values of 10.75 and 11.34 U, respectively. It can be clearly seen that the CS/PVA blend exhibited a continuous increase in POD activity until the end of the trial, where it recorded the significantly highest value of 14.34 U. In the case of PPO, the nonsignificant elevated values of activity in the early stage of ripening of CS/PVA/INA 0.5 mM and CS/PVA/INA 1.0 mM films (Figure 7B), followed by a significant reduction in the late stage of storage compared to control, indicated that both formulations had no adverse properties on the development of browning of tomato fruits. Meanwhile, the CS/PVA formulation showed the significantly highest activity of PPO after 15 and 20 days at 1.08 and 0.96 U, respectively.

As shown in Figure 7C, the PAL activity of CS/PVA fruits reached the significantly highest value on day 15 at 0.629 U and then decreased at the end of storage. The application of CS/PVA/INA 0.5 mM and CS/PVA/INA 1.0 mM treatments exhibited significantly lower values of PAL when compared with the control throughout the storage period. Figure 7D displays that CAT activity slowly increased in the early stage of ripening for coated and uncoated fruits, reaching the highest peak on the 15th day, before decreasing. The formulations of CS/PVA, CS/PVA/INA 0.5 mM, and CS/PVA/INA 1.0 mM increased CAT activity significantly throughout the experiment compared to the control uncoated group. The CS/PVA/INA 0.5 mM and CS/PVA/INA 1.0 mM formulations showed the significantly highest values of CAT at 7.29 and 7.48 U, respectively, on the 15th day of storage.

PAL is a crucial enzyme in the phenylpropanoid pathway, responsible for the biogenesis of phenolic constituents [65]. Enzymatic browning is a common phenomenon that can be noticed clearly in some fruits, which critically affects the quality attributes and the nutritive value. PPO induces the oxidation of polyphenolic constituents into their quinone derivatives, followed by further oxidization into the melanin pigment responsible for the browning reactions. Therefore, the activities of PAL and PPO mostly affected the TP content in fruits. However, the CS/PVA blend slightly induced browning enzymes, exhibiting the highest PAL and PPO activities on the 15th day of storage. There was no adverse effect on MDA content throughout the trial period, which could have been due to the huge accumulation of TPs that fight ROS. The activities of the PAL and PPO enzymes in the CS/PVA/INA 0.5 mM and CS/PVA/INA 1.0 mM treatments were always significantly lower than those of the control throughout the storage period (Figure 7C). The lower accumulation of TPs, along with the prevention of browning in tomatoes, in INA treatments seems logical. The activity of PPO is affected by the phenolic concentration and O_2_ concentration.

It was previously reported that nano-silicon in CS, but not CS only, is able to decrease the rate of the O_2_/CO_2_ transmission coefficient in tomato fruits [5]. Therefore, our study suggests that the linkage between CS and INA in coating films might confer the film with the ability to decrease the O_2_/CO_2_ transmission coefficient and consequently decrease the PPO activity and the browning of tomatoes. No studies have focused on the effect of INA on the browning enzymes of postharvest fruits. However, it was recently noted by Domiciano et al. [66] that rice leaves sprayed with picolinic acid (PiA), an isomer of INA, significantly increased PAL activity without any impact on PPO. On the contrary, a reduced PAL activity was noted in silicon/PiA treatment due to the physical barrier properties of the metal. The polymer blend of CS/PVA may act as a physical barrier to INA due to ionic interactions, thereby decreasing its diffusion into fruit tissue and, therefore, decreasing its impact on PAL activity. Likewise, a huge accumulation of PAL after 24 h of the application of INA at a higher concentration of 2.5 mM on tomato leaves was reported [16].

The antioxidant enzymes CAT and POD are found in plant tissue, and their activities reduce the formation of ROS [5]. In this study, the level of these enzymes was elevated under abiotic stress and progressively reduced when the operative stress recorded a huge rate of activity, thereafter inducing reactions related to the aging of the fruit, i.e., the loss of different nutrients and sensory features. The CS/PVA/INA 0.5 mM and CS/PVA/INA 1.0 mM treatments resulted in higher CAT activity in coated fruits than the control, demonstrating the role of these polymer films in retarding the senescence of tomatoes. In contrast, lower activities of POD in CS/PVA/INA 0.5 mM and CS/PVA/INA 1.0 mM treatments were noted in this study. This discrepancy in the pattern of antioxidant enzymes in polymer blends containing INA could have been due to the formation of low content of MDA related to oxidative stress, as well as a lower accumulation of the antioxidant phenolics in these treatments directly interfering with POD activity. Similarly, Zhu et al. [5] detected lower POD activity when studying the effect of nano-SiOx in chitosan on tomatoes harvested during the green stage. Furthermore, higher concentrations of INA were able to greatly induce the activity of POD and CAT antioxidant enzymes in tomato leaves after 24 h of treatment [16].

## 4. Conclusions

In this study, CS/PVA blends alone or containing INA were effectively established and applied to green tomato fruits to evaluate their protective effect under storage conditions. The coating polymer blend containing INA had UV-protective and antifungal properties. The outcomes obtained from this study displayed that tomato fruit coated with CS/PVA mixed with INA at a concentration of 0.5 and 1 mM had a significant effect on delaying pigmentation changes and TA, in addition to slowing the accumulation of TPs and, consequently, diminishing the activities of browning enzymes (PAL and PPO). Moreover, the CS/PVA/INA 0.5 mM and CS/PVA/INA 1.0 mM formulations induced the antioxidant enzyme CAT and diminished the production of MDA, thereby potentially protecting the cell membrane from degradation. This study proposes that low concentrations of INA in CS/PVA blends can not only extend the shelf-life of tomato (25–30%) but also increase the antioxidant capacity, mostly via CAT, along with minimizing browning enzymes. Furthermore, this study proposes that these blends are promising as biodegradable coatings for use in postharvest technology applications to increase shelf-life and preserve antioxidant activity in tomato fruits.

## Data Availability

All of the data are included in the article.
